# High resolution 3D diffusion cardiovascular magnetic resonance of carotid vessel wall to detect lipid core without contrast media

**DOI:** 10.1186/s12968-014-0067-z

**Published:** 2014-09-17

**Authors:** Yibin Xie, Wei Yu, Zhaoyang Fan, Christopher Nguyen, Xiaoming Bi, Jing An, Tianjing Zhang, Zhaoqi Zhang, Debiao Li

**Affiliations:** Biomedical Imaging Research Institute, Cedars-Sinai Medical Center, Los Angeles, CA USA; Department of Bioengineering, University of California, Los Angeles, CA USA; Department of Radiology, Anzhen Hospital, Capital Medical University, Beijing, China; MR R&D, Siemens Healthcare, Los Angeles, CA USA; MR Collaborations NE Asia, Siemens Healthcare, Beijing, China

**Keywords:** Atherosclerosis, Carotid, Vessel wall, Diffusion-weighted imaging, Lipid core, ADC, Plaque characterization, Cardiovascular magnetic resonance

## Abstract

**Background:**

Without the need of contrast media, diffusion-weighted imaging (DWI) has shown great promise for accurate detection of lipid-rich necrotic core (LRNC), a well-known feature of vulnerable plaques. However, limited resolution and poor image quality in vivo with conventional single-shot diffusion-weighted echo planar imaging (SS-DWEPI) has hindered its clinical application. The aim of this work is to develop a diffusion-prepared turbo-spin-echo (DP-TSE) technique for carotid plaque characterization with 3D high resolution and improved image quality.

**Methods:**

Unlike SS-DWEPI where the diffusion encoding is integrated in the EPI framework, DP-TSE uses a diffusion encoding module separated from the TSE framework, allowing for segmented acquisition without the sensitivity to phase errors. The interleaved, motion-compensated sequence was designed to enable 3D black-blood DWI of carotid arteries with sub-millimeter resolution. The sequence was tested on 12 healthy subjects and compared with SS-DWEPI for image quality, vessel wall visibility, and vessel wall thickness measurements. A pilot study was performed on 6 patients with carotid plaques using this sequence and compared with conventional contrast-enhanced multi-contrast 2D TSE as the reference.

**Results:**

DP-TSE demonstrated advantages over SS-DWEPI for resolution and image quality. In the healthy subjects, vessel wall visibility was significantly higher with diffusion-prepared TSE (p < 0.001). Vessel wall thicknesses measured from diffusion-prepared TSE were on average 35% thinner than those from the EPI images due to less distortion and partial volume effect (p < 0.001). ADC measurements of healthy carotid vessel wall are 1.53 ± 0.23 × 10^−3^ mm^2^/s. In patients the mean ADC measurements in the LRNC area were significantly lower (0.60 ± 0.16 × 10^−3^ mm^2^/s) than those of the fibrous plaque tissue (1.27 ± 0.29 × 10^−3^ mm^2^/s, p < 0.01).

**Conclusions:**

Diffusion-prepared CMR allows, for the first time, 3D DWI of the carotid arterial wall in vivo with high spatial resolution and improved image quality over SS-DWEPI. It can potentially detect LRNC without the use of contrast agents, allowing plaque characterization in patients with renal insufficiency.

**Electronic supplementary material:**

The online version of this article (doi:10.1186/s12968-014-0067-z) contains supplementary material, which is available to authorized users.

## Background

Stroke is a major worldwide health problem - every year it accounts for the death of an estimated 5 million people and leaves another 5 million permanently disabled [1]. Carotid artery atherosclerosis is a major cause of stroke and its subsequent disability and mortality [2]. Atherosclerotic plaques may cause stenosis of the arterial lumen resulting impaired cerebral perfusion. However, as a chronic and progressive disease, atherosclerosis remains asymptomatic in the majority of people due to the outward (positive) remodeling of the vessel wall. Therefore the degree of luminal stenosis alone is a relatively poor indicator of cerebral events [3,4]. Plaque disruption and rupture are believed to be more common etiology of cerebral ischemia and it is now widely accepted that greater emphasis should be placed on plaque composition characterization to determine its vulnerability [5,6]. Several histological studies from carotid endarterectomy specimens suggest that a typical culprit carotid plaque has a large lipid-rich necrotic core (LRNC), also known as “lipid core”, with a thin over-lying fibrous cap [5,7]. When the integrity of the fibrous cap is compromised, the lipid core is exposed to the blood-stream and thrombus formation and subsequent cerebral embolization may occur.

High-resolution multiple-contrast weighted cardiovascular magnetic resonance (CMR) has been used together with contrast enhancement (CE) to identify different plaque components with good histology correlation [8,9]. Gadolinium-based contrast media can preferentially enhance the fibrous cap providing the distinction from the LRNC, providing more accurate characterization than non-contrast T1- and T2-weighted imaging. Because CMR is noninvasive and does not involve ionizing radiation, it can be used clinically to monitor the progression of atherosclerotic disease and the outcome of therapeutic interventions [10]. However, several epidemiology studies have shown the association between atherosclerosis and chronic renal disease and many atherosclerosis patients have concomitant impaired renal function [11,12]. This makes the usage of CE CMR highly undesirable due to the increased risk of gadolinium-associated nephrogenic systemic fibrosis [13]. Moreover, the added cost and procedural complexity of CE reduces the appeal of CMR as a potential tool for disease screening in large population and longitudinal evaluation of therapeutics.

As a non-contrast alternative, diffusion-weighed imaging (DWI) with apparent diffusion coefficient (ADC) mapping has showed great promises for carotid plaque characterization, with excellent image contrast for discriminating the LRNC from the surrounding fibrous tissue. For example Qiao et al. reported that DWI can selectively identify LRNC with high contrast and accuracy, whereas conventional T1- and T2-weighted images, by comparison, do not accurately identify LRNC [14]. Clarke et al. compared eight MR contrast weightings including T1w, T2w, PDw etc., and found diffusion weighting was the only image contrast in which the signal standard deviation for LRNC and fibrous tissue did not overlap [15]. Two other groups applied DWI in vivo and achieved good correlation with histology for the detection of LRNC [16,17]. However, to our knowledge, all in vivo DWI studies so far were based on 2D single-shot diffusion-weighted echo-planar imaging (SS-DWEPI). It is a time-efficient sequence and available on all major commercial systems, yet is known to suffer from suboptimal image quality with susceptibility-induced image distortion, dropout, blurring, and signal loss. In carotid imaging applications, the air/tissue and bone/tissue interfaces around the cervical spine region have large susceptibility differences therefore are especially challenging for SS-DWEPI acquisitions. Moreover, SS-DWEPI provides very limited spatial resolution (typical in-plane pixel size ≥1.0 × 1.0 mm^2^). Attempts to increase spatial resolution will further deteriorate image quality with more T2* decay during the course of SS-DWEPI. When imaging fine anatomy such as atherosclerotic plaques, where the regions-of-interest (ROIs) typically contains only a few pixels, conventional SS-DWEPI will likely cause severe partial volume effects and inaccuracies in plaque ADC measurements [14,18].

The purpose of this work is to develop a novel DWI method for non-contrast carotid plaque characterization that provides three major improvements over current 2D SS-DWEPI: (1) 3D imaging capability; (2) high spatial resolution (0.6 × 0.6 × 2 mm^3^); and (3) reliable image quality.

## Methods

### Pulse sequence design

**Figure 1:**
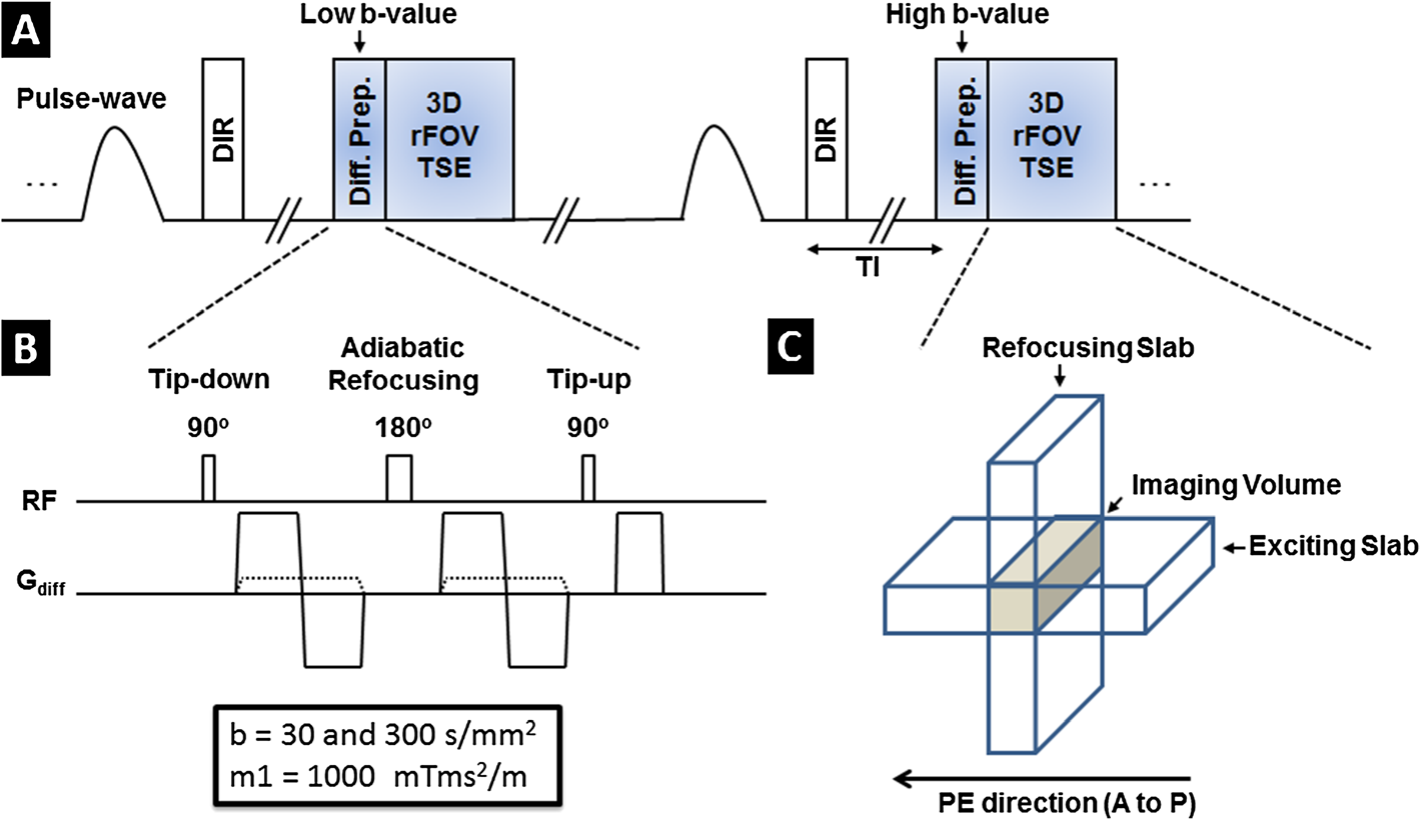
**Imaging sequence design of black-blood DP-TSE with rFOV. (A)** The pulse-triggered data acquisition scheme consists of a black-blood (DIR) preparation module, a diffusion-preparation module, and a segmented 3D TSE kernel with reduced field-of-view. Two diffusion-weighted images were acquired in an interleaved fashion to minimize the mismatch between them. **(B)** Diffusion preparation module. A pair of bipolar gradients was used for diffusion encoding with complete compensation for first-order motion. Adiabatic refocussing pulse was used for its insensitivity to b1-inhomogeneity. In order to suppress residual arterial blood, additional flow-dephasing gradients were incorporated into the module with moderate first gradient moment of 1000 mTms^2^/m at both diffusion weightings. **(C)** Illustration of the reduced field-of-view scheme. Excitation slab is perpendicular to the slice direction whereas the refocussing slab was oriented to be perpendicular to the phase direction, limiting the imaging volume to the overlapping region between the two slabs (brown region). Because of the reduced imaging volume, phase encoding steps could be greatly reduced to shorten scan time.

The MR pulse sequence development contained the design and implementation of two major parts: a motion-compensated diffusion preparation module and a reduced field-of-view (rFOV) 3D turbo spin echo (TSE) readout (Figure 1). The diffusion preparation module was implemented based on a driven equilibrium (DE) preparation [19,20], of which the variants are commonly used in dark-blood imaging [21,22]. Bipolar diffusion sensitizing gradients were used to compensate for 1st-order motion and to reduce eddy currents. Additional specifications of the preparation module are included in Table 1. Acquisitions of different diffusion weightings were interleaved in order to minimize the mismatch between images. Pulse-triggering was utilized to synchronize the sequence to each subject’s cardiac rhythm in order to minimize arterial pulsatile motion. Imaging readout was implemented based on a 3D TSE kernel for reliable image quality at 3 T and high SNR. Reduced field-of-view was developed to reduce scan time with inner-volume refocusing pulses [23]: gradients of the refocusing pulses in TSE were moved from the slice-encoding direction to the phase-encoding direction, with their magnitude modified accordingly based on the desired rFOV size. Arterial blood suppression scheme was designed with a combination of double inversion recovery (DIR) and flow-sensitive dephasing (FSD) [24] in order to effectively suppress flow artifacts, improve vessel wall visualization and reduce partial volume effect.

**Table 1:** **Sequence parameters of DP-TSE and SS-DWEPI used in this study**

	**DP-TSE**	**SS-DWEPI**
Acquisition type	3D	2D
In-plane resolution (mm^2^)	0.6 × 0.6	1.2 × 1.2
Number of slices	12	12
Slice thickness (mm)	2.0	2.0
TR (s)	2RR	4.3
TE (ms, same for both b-value)	42 (effective)	75
Matrix	256 × 76	128 × 128
Partial Fourier	No	0.75
Field of view (mm)	160 × 47	160 × 160
ETL	12	-
BW (Hz/pixel)	130	1002
NEX	2	32
*b* value (s/mm^2^)	30/300	50/300
Fat suppression	CHESS	CHESS
Max diffusion grad. (mT/m)	43	Default (25)
Max FSD grad. (mT/m)	2.2	N/A
Diffusion grad. direction	All three axes	Slice
Scan time	~5′30″	5′24″

### In vivo imaging

With informed consent and approval from internal review board (IRB), healthy volunteers (n = 15; 5 M, 10 F; aged 23–48 y/o) and patients with diagnosed or suspected carotid atherosclerosis (n = 6; 4 M, 2 F; aged 58–81 y/o) were recruited and scanned on a 3 T scanner (MAGNETOM Verio; Siemens AG, Erlangen, Germany) with a 4-channel carotid coil (Machnet BV, Roden, The Netherlands). Scanning parameters included 3D transverse slab of 12 slices with in-plane resolution of 0.6 × 0.6 mm^2^ and slice thickness of 2 mm. Two diffusion weighted images with *b* values of 30 and 300 s/mm^2^ were acquired in an interleaved fashion. FSD with first gradient moment of 1000 mTms^2^/m was used for blood suppression in combination with conventional double inversion recovery (DIR) preparation. Other details of this protocol are summarized in Table 1.

Healthy volunteers were also scanned using conventional SS-DWEPI for comparison. Due to the limitations of SS-DWEPI, lower in-plane resolution (1.2 × 1.2 mm^2^) were used along with 75% partial Fourier in the phase direction. Diffusion weightings of b = 50 and 300 s/mm^2^ along the slice direction were acquired. Other parameters are also listed in Table 1. This protocol was adapted for the scanner based on previously published studies by other groups [17,18].

Patients underwent additional clinical scans of pre-contrast T2-weighted imaging as well as pre- and post-contrast enhanced T1-weighted imaging as the reference using a conventional 2D TSE protocol similar to the ones described by previous studies [8,9]. Common imaging parameters include: FOV = 160 × 160 mm^2^; in-plane resolution = 0.6 × 0.6 mm^2^; slice thickness = 2.0 mm; and TR/TE = 720/9.4 (T1w), 4000/60 (T2w).

### Image processing and evaluation

#### SNR, CNR and ADC measurement

Vessel wall SNR and CNR were evaluated using region of interest (ROI) analysis on both of the diffusion weighted images. Measurements were performed using image analysis toolbox in MATLAB (ver. 2011, Mathworks, Natick, MA). For each 3D volume, three image slices from the center to the peripheral of the slab were analyzed. ROIs of vessel wall and lumen from both sides of the carotid arteries were manually contoured and the signal intensities (S_wall_, S_lumen_) are recorded. Noise level (S_noise_) was defined as standard deviation within ROIs drawn in peripheral air space of the image uncontaminated by artifacts. The relative vessel wall SNR and CNR were defined as:

$$ \mathrm{SNR}={\mathrm{S}}_{\mathrm{wall}}/{\mathrm{S}}_{\mathrm{noise}};\kern1em \mathrm{CNR}=\left({\mathrm{S}}_{\mathrm{wall}}-{\mathrm{S}}_{\mathrm{lumen}}\right)/{\mathrm{S}}_{\mathrm{noise}} $$

ADC of normal carotid wall was calculated from the ADC map using similar ROI analysis. ADC of LRNC and fibrous plaque tissue in patients was measured based on the ROIs identified on the post-contrast enhanced T1-weighted image of the two types of tissue, respectively.

#### Wall visibility

Vessel wall visibility was quantified on both DP-TSE and SS-DWEPI images by three independent reviewers (ZF, CN and XB) who are blinded to the image type. Three DWI (300 s/mm^2^) images at the level of common carotid arteries were evaluated from each subject. In total 90 common carotid vessel walls from the 15 healthy subjects were rated based on the fractions of vessel wall visible ranging from 0% to 100%. The vessel wall visibility grading was averaged among the reviewers and then classified to five groups: less than 20% (not visible); 20%-50% (poor); 50%-75% (good); 75-95% (excellent); more than 95% (complete). A histogram was then generated of the counts of vessel walls with different levels of wall visibility for DP-TSE versus SS-DWEPI.

#### Wall thickness

In order to evaluate the partial volume effect, a computer assisted morphometric analysis of vessel wall was performed on a workstation using Image-Pro Premier (Media Cybernetics, Rockville, MD). This software performed semiautomatic tracing of the vessel wall inner and outer boundaries and calculated the distance between them, of which the mean was defined as wall thickness. Two common artery vessel walls were measured from each of the subjects on DP-TSE image, SS-DWEPI image, and conventional anatomical image (T2-weighted 2D TSE).

#### Statistical analysis

Seven slices with LRNC were identified in three patients using CE T1w images as the reference. ROIs of LRNC, fibrous plaque tissue and normal vessel were defined in CE T1w images and copied to DWI and ADC images for quantification. Slight translational adjustments of the ROIs were made in the cases where inter-scan movement was observed. In total, 265 pixels of LRNC, 289 pixels of fibrous plaque tissue, and 356 pixels of normal vessel wall from DP-TSE images were included in the calculation and the global mean and standard deviation of ADC values were computed. No normalization was used on ADC values between subjects and different tissue types. An unbalanced one-way ANOVA was performed on the mean ADC values of LRNC, fibrous plaque tissue, and adjacent normal vessel wall at each plaque location after verification of normality with quantile-quantile plot and Shapiro-Wilk test. Paired Student’s t-tests were performed on the vessel wall visibility comparison and vessel wall thickness comparison between DP-TSE and SS-DWEPI. All statistical analysis was performed in R statistical programming language (ver. 3.0.3, The R Foundation for Statistical Computing, Vienna, Austria).

## Results

**Figure 2:**
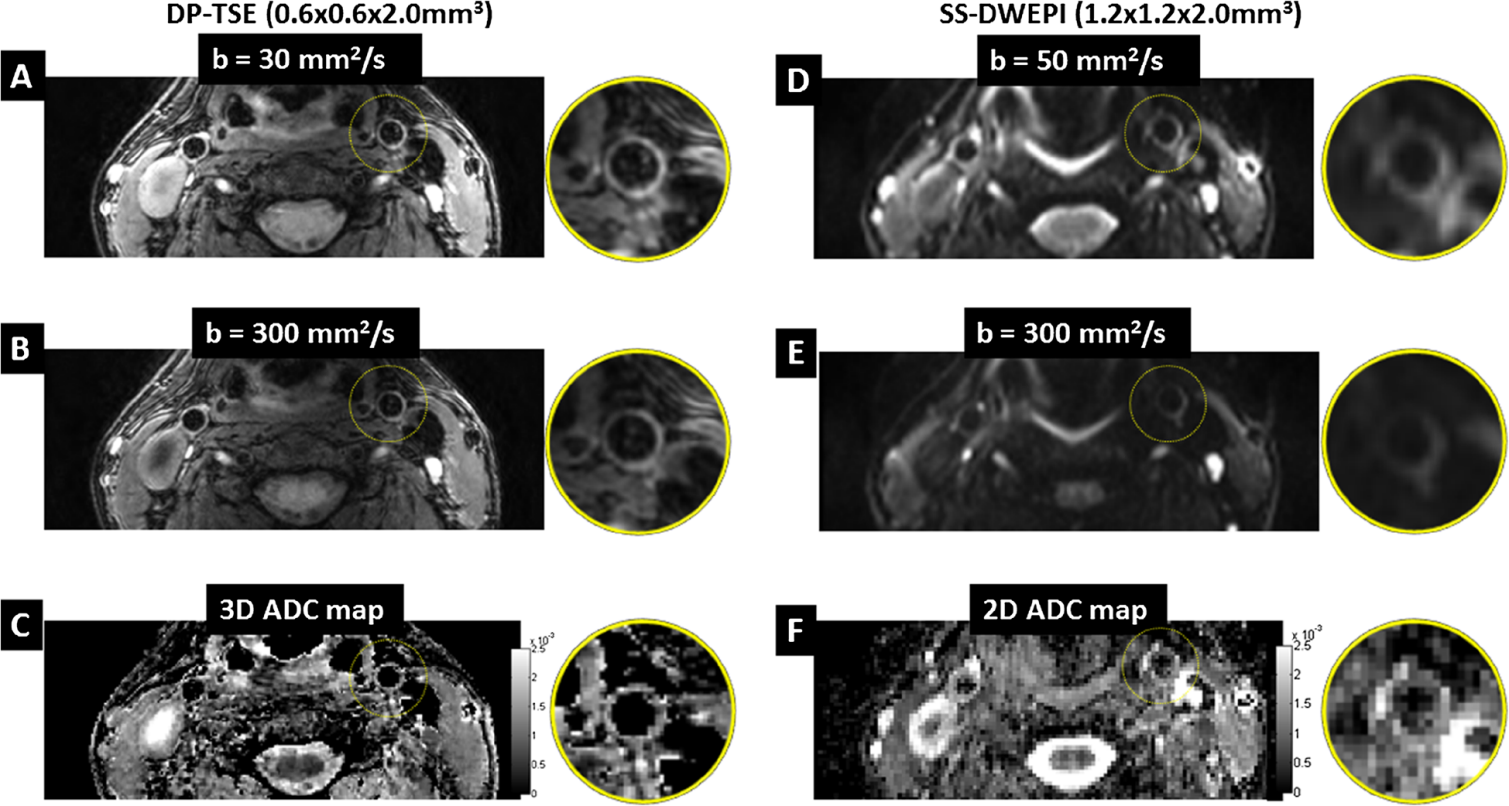
**Representative image quality of 3D DP-TSE compared with that of conventional 2D SS-DWEPI from a healthy subject. (A to C)** DWI of b = 30 mm^2^/s, DWI of b = 300 mm^2^/s and ADC map, respectively, acquired using DP-TSE with resolution of 0.6 × 0.6 × 2.0 mm^3^. **(D to F)** DWI of b = 50 mm^2^/s, DWI of b = 300 mm^2^/s and ADC map, respectively, acquired using SS-DWEPI with resolution of 1.2 × 1.2 × 2.0 mm^3^. Note that blurring, distortion and artifacts were present in the SS-DWEPI images compared with their counterparts from DP-TSE. Also note that SS-DWEPI images had higher T2 decay due to the longer required TE than that of the proposed DP-TSE.

In each of the 21 subjects, the proposed DP-TSE method showed improved visualization of carotid vessel wall than SS-DWEPI, in which signal loss and distortion were commonly observed. The increase in spatial resolution and reduction of image artifacts, especially distortion, yielded markedly sharper vessel wall images in DP-TSE images. Figure 2 shows a representative case of a healthy subject comparing the image quality of DP-TSE, on the left, versus SS-DWEPI, on the right, at two b-values and the resultant ADC maps. Figure 2A and B are representative DP-TSE images of carotid vessel wall from healthy subjects at b = 30 and 300 s/mm^2^, respectively. Arterial blood suppression was effective throughout the slices with clear visualization of vessel wall at both b-values. No visible susceptibility-induced artifacts were observed. ADC map at the corresponding slice showed clear, complete vessel wall (Figure 2C).

**Figure 3:**
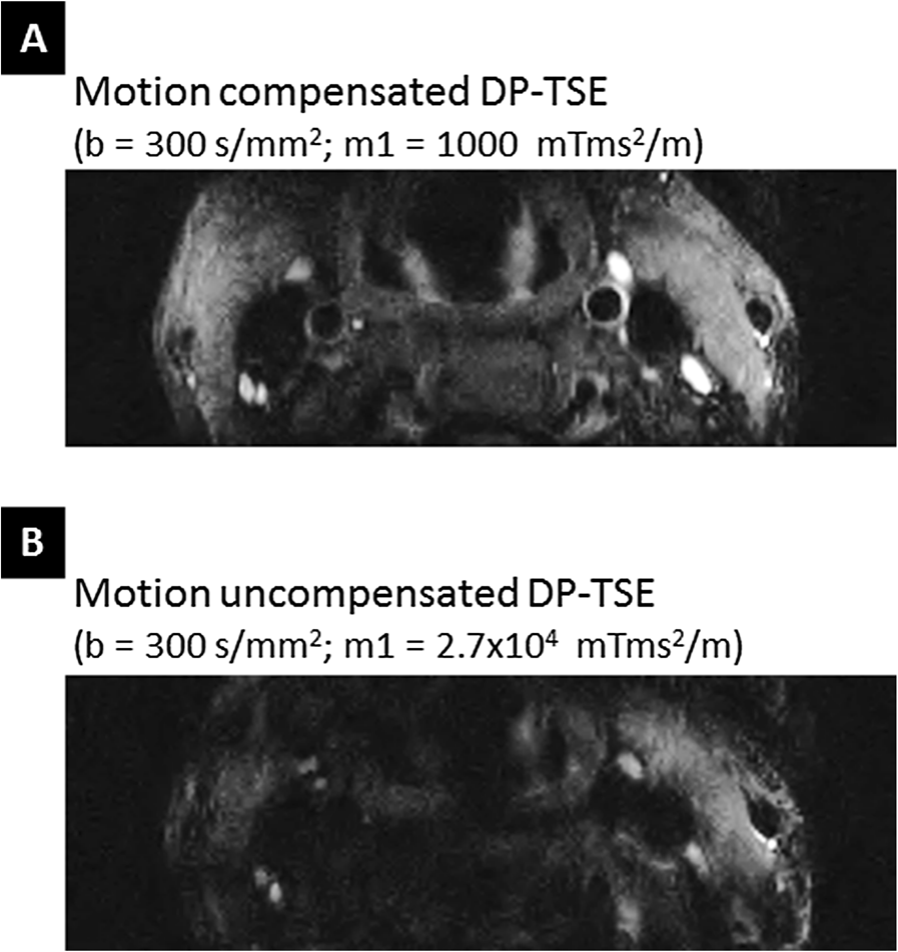
**Effectiveness of motion compensation in diffusion preparation.** At the same b-value, motion compensated preparation **(A)** preserved the vessel wall signal well whereas the uncompensated preparation **(B)** resulted in major loss of signal in the vessel wall due to the large first order gradient moment. A pulse wave-gated cine of the carotid vessel wall is presented [see Additional file 1] to demonstrate the pulsatile motion in the carotid artery.

There was no apparent signal loss due to motion in the DP-TSE images which suggested that 1st-order motion compensation was effective. Figure 3 demonstrates the effects of motion compensation in the diffusion preparation with a representative case. At the same b-value, motion compensated preparation (Figure 3A) preserved the vessel wall signal well whereas the uncompensated preparation (Figure 3B) resulted in major loss of signal in the vessel wall due to the large first order gradient moment. A pulse wave-gated cine of the carotid vessel wall is presented to demonstrate the pulsatile motion in the carotid artery caused by arterial flow [see Additional file 1].

**Figure 4:**
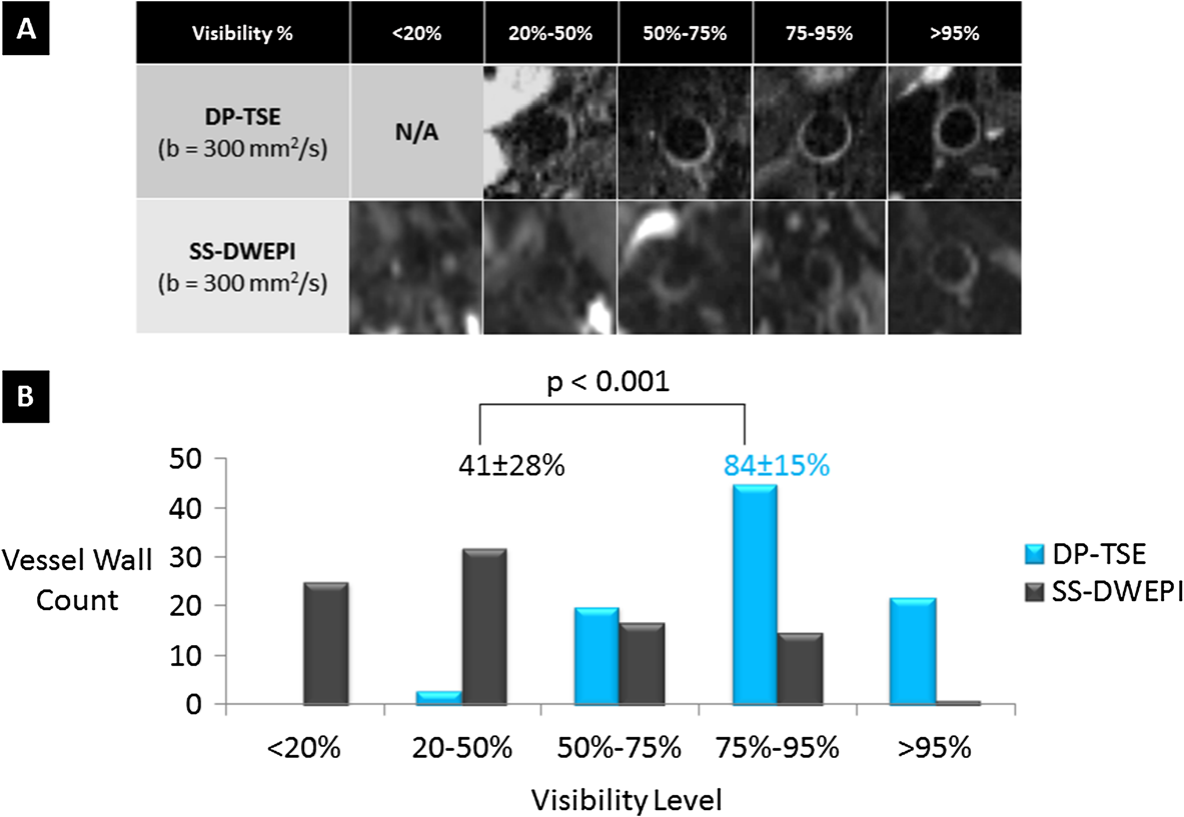
**Vessel wall visibility comparison between 3D DP-TSE and conventional 2D SS-DWEPI. (A)** Representative diffusion-weighted images from the DP-TSE and SS-DWEPI with different levels of visualization ranging from 0% to 100%. **(B)** Vessel wall visibility histograms of DP-TSE and SS-DWEPI. DP-TSE displayed a higher distribution and significantly higher average visibility than SS-DWEPI.

Vessel wall visibility was significantly better with DP-TSE. Figure 4A shows representative images from DP-TSE and SS-DWEPI with different levels of visualization ranging from 0% to 100%. The histogram of the vessel wall visibility shows that the distribution of DP-TSE lied towards the higher end of the chart than that of SS-DWEPI (Figure 4B). The average wall visibility of DP-TSE images was 84 ± 15%, significantly higher than 41 ± 28% of SS-DWEPI images (p < 0.001).

Vessel wall SNR and CNR quantified by ROI analysis was summarized in Table 2. Satisfactory vessel wall SNR provided further confirmation that there was no major signal loss due to motion. The vessel wall to lumen CNR suggested that the arterial blood suppression was effective.

**Table 2:** **SNR, CNR measurements of carotid vessel walls in DP-TSE images**

**Image quantification**	**b = 30 (s/mm** ^**2**^ **)**	**b = 300 (s/mm** ^**2**^ **)**
Mean SNR	14.9 ± 2.8	11.6 ± 2.1
Mean CNR	13.2 ± 2.6	9.9 ± 1.9

**Figure 5:**
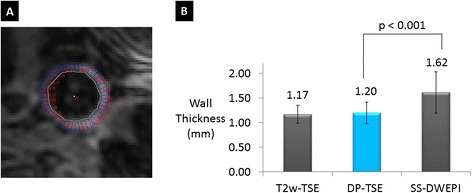
**Computer-assisted morphometric measurements of vessel wall thickness. (A)** Representative vessel wall thickness measurements on a DP-TSE image. Vessel wall inner and outer boundaries are traced and wall thicknesses are calculated along these traces using a semiautomatic program. **(B)** Vessel wall thicknesses measured from DP-TSE were significantly thinner (35% on average) than those from the EPI images due to less distortion and partial volume effect.

DP-TSE provided more accurate wall thickness measurements than SS-DWEPI. Figure 5B shows that the mean vessel wall thickness measured from the proposed method was close to the reference anatomical images, which was 35% thinner than SS-DWEPI images (p < 0.001) due to less image distortion and less partial volume effect.

**Figure 6:**
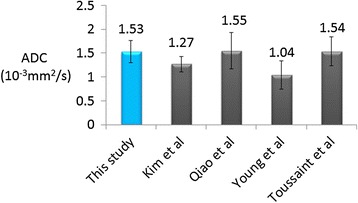
**ADC measurements of normal carotid wall from 3D DP-TSE images.** The value is comparable with previous in vivo (Kim, Young) and ex vivo (Qiao, Toussaint) studies.

The ADC measurement of carotid vessel wall in healthy subjects from this study was 1.53 ± 0.23 × 10^−3^ mm^2^/s, which was comparable with previous in vivo and ex vivo studies (Figure 6).

**Figure 7:**
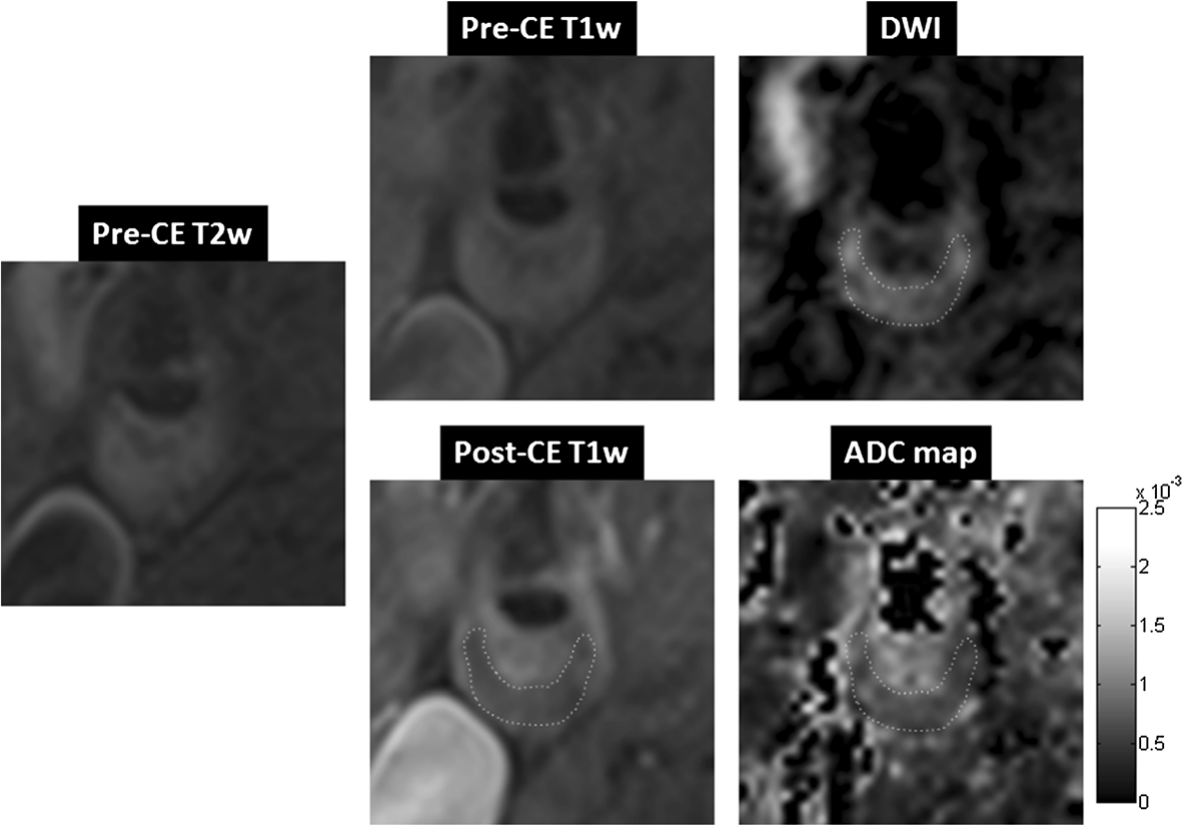
**A case study of a symptomatic subject with an atherosclerotic plaque of 70% stenosis in the right internal carotid artery.** Pre-contrast T2-weighted image showed slightly hyper-intense signal throughout the plaque area. Pre-contrast T1-weighted image was iso-intense in the plaque area. Post-contrast T1-weighted image showed a clear hypo-intense area within the plaque, a typical LRNC appearance, surrounded by enhanced fibrous plaque tissue. DWI (b = 300 mm^2^/s) using DP-TSE showed a hyper-intense region, i.e. low diffusion, that spatially matched to the LRNC area in the post-contrast T1-weighted image. ADC map showed an area with low diffusion (0.62 ± 0.15 × 10^−3^ mm^2^/s) within the plaque that spatially matched to the LRNC finding in the post-contrast T1-weighted image.

**Figure 8:**
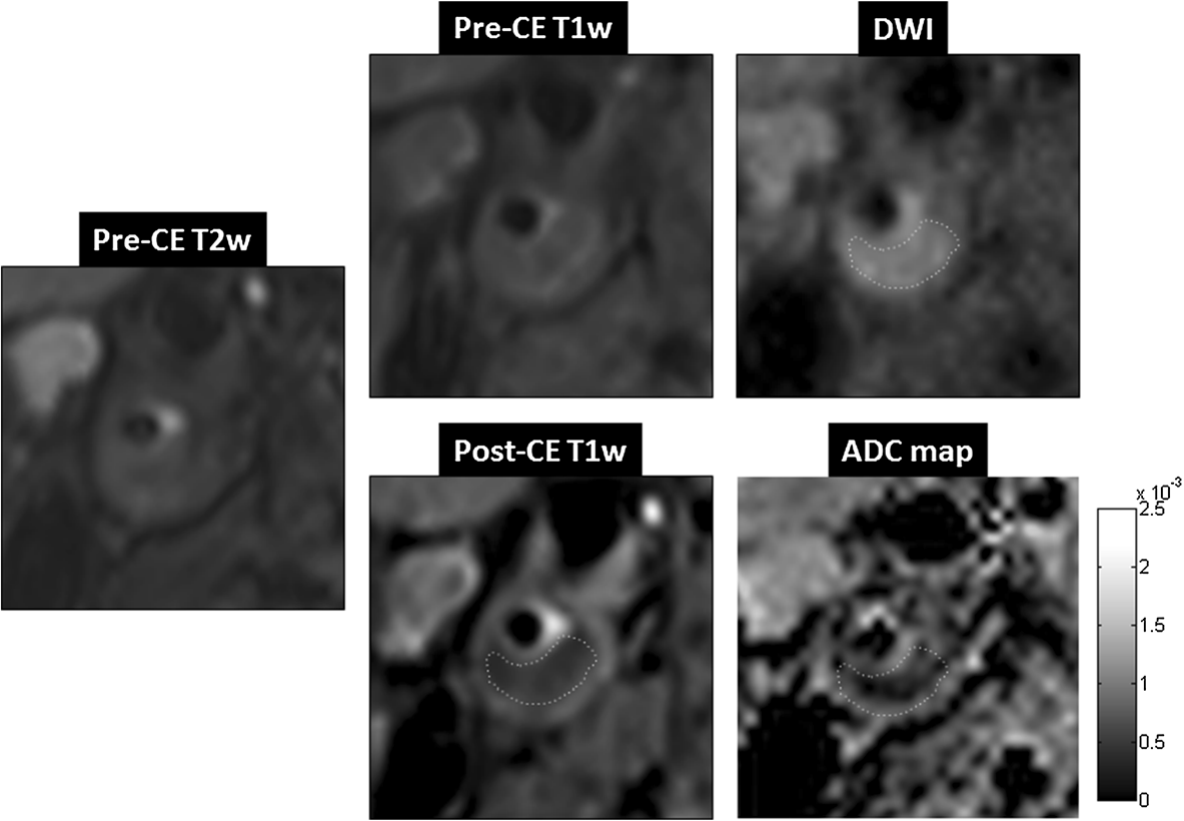
**A case study of a symptomatic subject with an atherosclerotic plaque of 80% stenosis in the right internal carotid artery.** Pre-contrast T2-weighted image and T1-weighted image were both iso-intense in the plaque area. Post-contrast T1-weighted TSE image showed a hypo-intense area within the plaque surrounded by enhanced tissue, indicating a large LRNC. DWI (b = 300 mm^2^/s) using DP-TSE shows a hyper-intense region, i.e. low diffusion, that spatially matched to the LRNC area in the post-contrast T1-weighted image. ADC map showed an area with low diffusion (0.46 ± 0.22 × 10^−3^ mm^2^/s) within the plaque that also spatially matched to the LRNC finding in the post-contrast T1-weighted image.

Excellent agreement was observed between DP-TSE images (both DWI and ADC map) and conventional CE CMR in identifying LRNC. Two representative studies are presented in Figures 7 and 8. In both cases pre-contrast T1-weighted and T2-weighted TSE images provided little diagnostic information on the plaque composition. Post-contrast T1-weighted image showed a clear hypo-intense area within the plaque surrounded by enhanced fibrous plaque tissue, indicating LRNC. DWI (b = 300 s/mm^2^) showed an area with reduced diffusion (hyper-intense) which matched to the area in the post-contrast T1-weighted image with low contrast uptake (hypo-intense). The resultant ADC map also confirmed that the area with low diffusion within the plaque was spatially matched to the LRNC region in the post-contrast T1-weighted image.

**Figure 9:**
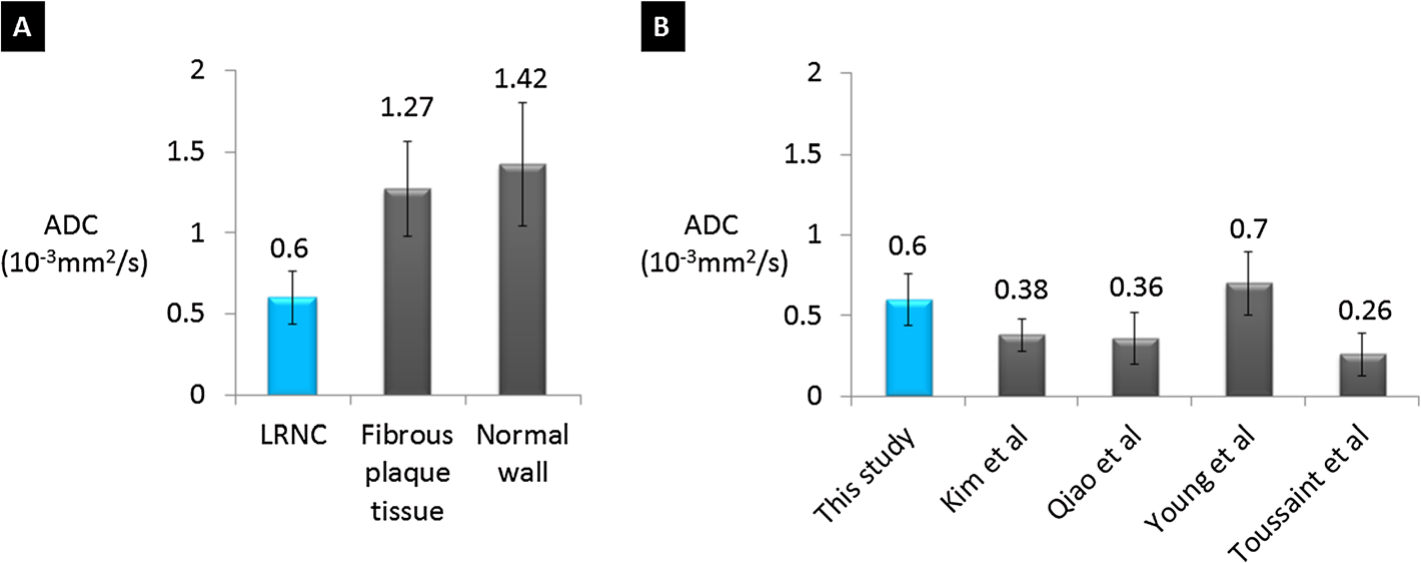
**ADC measurements of LRNC from 3D DP-TSE images and comparison. (A)** The mean ADC measured from DP-TSE images in LRNC areas was significantly lower (0.60 ± 0.16 × 10^−3^ mm^2^/s) than that of the fibrous plaque tissue (1.27 ± 0.29 × 10^−3^ mm^2^/s) and normal wall tissue (1.42 ± 0.38 × 10^−3^ mm^2^/s) with p < 0.01. **(B)** The mean ADC of LRNC measured from DP-TSE images in this study compared with results from previous in vivo (Kim, Young) and ex vivo (Qiao, Toussaint) studies.

The ADC measurements in plaques based on region-of-interest analysis revealed markedly reduced apparent diffusion in LRNC compared with fibrous plaque tissue and normal vessel wall (Figure 9). The mean ADC of LRNC (265 voxels) was 0.60 ± 0.16 × 10^−3^ mm^2^/s, whereas the mean ADC of fibrous plaque tissue (289 voxels) and normal vessel wall (356 voxels) was 1.27 ± 0.29 × 10^−3^ mm^2^/s and 1.42 ± 0.38 × 10^−3^ mm^2^/s, respectively. The one-way ANOVA showed significant difference between the mean values (F (2,20) = 79; p < 0.01).

## Discussion

A 3D DP-TSE technique was developed for high-resolution DWI of the carotid artery wall. With comparable imaging time, the proposed method provided four-times better spatial resolution (0.6 × 0.6 × 2 mm^3^ for DP-TSE vs 1.2 × 1.2 × 2 mm^3^ for SS-DWEPI), markedly improved wall visibility, and more accurate wall thickness compared with SS-DWEPI in healthy volunteer studies. In contrast, signal loss and distortion were commonly seen in the conventional SS-DWEPI images, an observation consistent with the previous in vivo study by Kim et al. [18]. In patient studies, excellent agreement was observed between DP-TSE and conventional CE CMR in identifying LRNC. ADC measurements in LRNC were significantly different from those of the fibrous plaque tissue and normal vessel wall.

DP-TSE allowed, for the first time, 3D DWI of the carotid arterial wall in vivo with sub-millimeter spatial resolution and excellent image quality on 3 T. This was made feasible by the sequence design of a separated diffusion encoding module from the imaging readout, therefore enabling segmented acquisition. Unlike multi-shot EPI which is usually sensitive to phase errors [25], the separated driven-equilibrium diffusion preparation module tipped up the magnetization to the longitudinal axis and spoiled the residual transverse magnetization at the end of the preparation to eliminate phase dispersion. It was therefore less vulnerable to phase inconsistency between shots. Essentially, diffusion preparation only modulated the longitudinal magnetization to introduce diffusion-weighting. For the same reason, it did not violate the CPMG condition required by the following TSE readout kernel.

Imaging readout was developed based on TSE with reduced field-of-view. TSE is known to be relatively robust to field inhomogeneity [26], therefore adopted in this work for imaging carotid arteries at 3 T. Inner-volume reduced field-of-view method was used to reduce phase encoding steps therefore shortening scan time. The disadvantage of rFOV is the SNR reduction associated with reduced imaging volume. However, even with rFOV, 3D TSE had approximately four-times the excited volume as conventional 2D TSE carotid imaging with comparable scan time.

The diffusion preparation design focused on two important aspects to cater to carotid vessel wall imaging: blood suppression and motion compensation. Effective arterial blood suppression (black-blood) was required to eliminate flow-induced artifacts commonly seen in carotid vessel wall imaging. Additionally, black-blood improved vessel wall visibility and eliminated blood pool partial volume effects at the blood/wall interface. In this study, the diffusion preparation and imaging readout were gated to the diastole in order to minimize the effects of motion. The slow flow in diastole and thick 3D imaging slab made black-blood imaging more challenging than conventional ungated 2D imaging. To address this, black-blood was achieved using the synergy of DIR and FSD [24]. DIR was utilized prior to the diffusion preparation when faster systolic inflow occurs. FSD is integrated into the non-selective diffusion preparation to dephase the residual blood spins throughout the imaging slab, therefore relaxing the requirement of complete inflow on DIR. Bipolar diffusion encoding gradients were used in all three directions to null the first order gradient moment, making the preparation relatively insensitive to motion. Additional FSD gradients were applied only in the slice direction, where flow was at its maximum and pulsatile motion was at its minimum [27].

ADC measurements of the healthy carotid vessel wall, LRNC, and fibrous plaque tissue in this study were comparable with those reported in previous in vivo and ex vivo studies [14,16,17,28]. This provided preliminary support for the proposed new DWI method. However further validation is mandatory due to the limited magnitude of the patient study and the lack of histology in this technical work. With the indications from these preliminary results, double-blinded readings of DWI and CE CMR shall be performed and compared with histological results independently in a larger patient population in the future.

As a quantitative biomarker, ADC could potentially provide a more objective measure of the plaque lipid content. Young et al. have shown that ADC can demonstrate not only the presence of lipid but also the amount [17]. ADC may serve as a surrogate marker for longitudinal studies evaluating the effectiveness of lipid-lowering therapies and other medical treatments. As a similar observation we noticed in this study that the mean ADC from atherosclerosis patients was lower than that from healthy subjects even for normal vessel wall. We hypothesize that even with no visible plaque, the ‘normal wall’ in patients may have lipid infiltration that caused the reduction of ADC. However it is important to note that although significantly lower ADC values have been observed in LRNC, the reference ADC ranges for healthy wall and LRNC in vivo have not been established. The limited resolution and image quality issues with conventional SS-DWEPI have been a major obstacle and the proposed method may serve as a step towards more accurate plaque diffusion quantification. Lastly, carotid plaque characteristics reflect generalized atherosclerosis [29] and the composition of carotid plaques determined by DWI may reveal the vulnerability of atherosclerotic plaques elsewhere.

## Conclusions

DP-TSE is a promising method for 3D diffusion imaging of carotid vessel wall with high spatial resolution and improved image quality over SS-DWEPI. It provides a potential means to detect LRNC in carotid plaque in vivo without the use of gadolinium-based contrast media, allowing carotid plaque characterization in patients with renal insufficiency.

## Additional file

Additional file 1:
**Pulse wave-gated cine of the carotid vessel wall demonstrating arterial pulsatile motion.** A retrospectively pulse wave-gated cine of the carotid vessel wall demonstrates the pulsatile motion in the carotid artery caused by arterial flow (acquired spatial resolution = 0.55 × 0.55 × 4 mm^2^; temporal resolution = 22.6 fps; FLASH readout).
